# Safety of ultra‐low contrast coronary angiography in patients with acute kidney injury

**DOI:** 10.1002/clc.24282

**Published:** 2024-05-27

**Authors:** Zach Rozenbaum, Mailing Flores Chang, Jose Wiley, Ali Gholam, Anand Irimpen, Ali A. Alsaad

**Affiliations:** ^1^ Department of Cardiology Tulane University New Orleans Louisiana USA

**Keywords:** acute renal failure, AKI, contrast‐induced nephropathy, coronary angiography, ultra‐low contrast

## Abstract

**Background:**

Ultra‐low contrast administration during coronary angiography has been previously shown to be feasible and safe among patients with stable chronic kidney disease. In the present study, we investigate the safety of ultra‐low contrast coronary angiography in patients with pre‐existing acute kidney injury (AKI).

**Methods:**

The study was a retrospective single‐center evaluation of hospitalized patients who had AKI and required coronary angiography. Ultra‐low contrast use was defined as ≤18 mL of contrast media.

**Results:**

The cohort consisted of a case series of eight inpatients with AKI who required coronary angiography. The mean age was 57 (±16) years and half were females. All patients had chronic kidney disease with a mean baseline estimated glomerular filtration rate of 34 (±17) mL/min/1.73 m^2^. The mean creatinine before angiography was 3 (±1) mg/dL and volume of contrast administered was 14 (±4) mL. One patient had a 0.1 mg/dL increase in creatinine during admission, and no patients had further AKI up to 1‐week postprocedure.

**Conclusions:**

The current data suggest that ultra‐low contrast coronary angiography can be safely performed in patients with pre‐existing AKI The study should be viewed as hypothesis‐generating due to its small sample size. A larger cohort is required to validate the results.

## Graphical abstract

Eight patients who had acute kidney injury (AKI) and an indication for coronary angiography were included. After 24 h of stabile serum creatinine, patients underwent ultra‐low contrast angiography. None of the participants had further AKI postprocedure. This hypothesis‐generating small sample study suggests that ultra‐low contrast coronary angiography can be safely performed in patients with pre‐existing AKI.

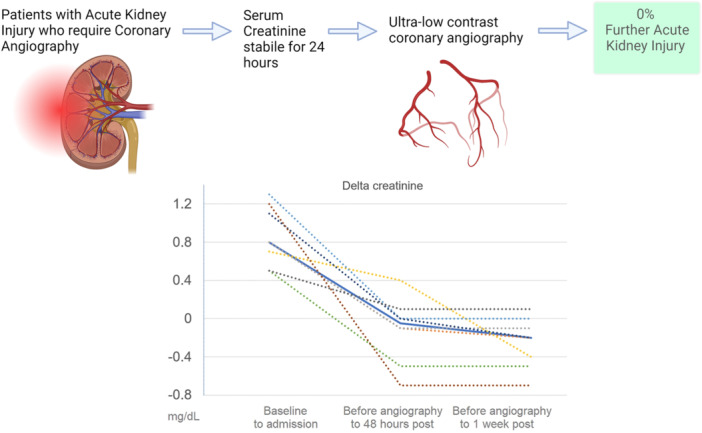

## 1 INTRODUCTION

Contrast‐induced nephropathy (CIN) remains a major source of morbidity in contemporary invasive cardiology practice. There are several well‐established risk factors,^1^ including higher amounts of contrast agent administration during coronary angiography,^2^ and chronic kidney disease (CKD).^3^ When CIN occurs, it is associated with adverse outcomes.^4^, ^5^ Therefore, various measures for prevention of CIN have been explored.^1^ Hydration protocols were shown to reduce the risk of CIN.^6^, ^7^ Reducing volumes of contrast administration is also effective in preventing CIN.^8^ Accordingly, methods for ultra‐low use of contrast agents during coronary angiography were developed^9^ and shown to be feasible and safe.^10^ Previous studies evaluating ultra‐low contrast coronary angiography and interventions included patients with CKD.^8^, ^10^, ^11^ The techniques have not been evaluated on patients with acute kidney injury (AKI). In the present study, we investigate the safety of ultra‐low contrast coronary angiography in patients with pre‐existing AKI.

## 2 METHODS

### 2.1 Population and definitions

The study was a case series from a single‐center of patients with AKI who required coronary angiography over a 12‐month period during 2022–2023. AKI was defined as an increase in serum creatinine (SCr) by ≥50% within 7 days or an increase in SCr by ≥0.3 mg/dl within 2 days.^12^ Baseline sCr levels were measured either on admission (*n* = 6) or on available laboratory tests during the week before the angiography (*n* = 2). The baseline sCr level was compared to the sCr level on the day of the angiography. All patients had stable sCr within the 24 h before angiography. Patients with AKI in whom the application of ultra‐low contrast techniques was not practical, such as shock, ST‐elevation myocardial infarction, respiratory failure, or hemodynamic instability, were not included in the analysis. Four hundred and twenty coronary angiographies were screened and 24 patients who clearly had AKI and had undergone coronary angiography without ultra‐low contrast coronary angiography techniques were excluded. Ultra‐low contrast coronary angiography was defined as administration of 18 ml of contrast or less during the index procedure. Methods for ultra‐low contrast coronary angiography were previously described.^7^, ^9^ In short, coronary artery engagement was performed using conventional coronary catheters and saline injections or guidewire‐facilitated engagement. Large caliber catheters were avoided, and contrast was aspirated before catheter exchange. Contrast was diluted by approximately 30% with saline and cine frame rate was increased. Limited projections were performed and if further characterization of lesions was required intravascular ultrasound and/or instantaneous wave‐free ratio were used. Management of renal dysfunction was left to the discretion of the primary team and guided by the etiology of AKI. There was no post‐hydration protocol due to the low amount of contrast administration.

Levels of sCR were documented at 24, 48, and 72 h after the angiography. CIN was defined as an increase in 0.5 mg/dL, or a relative rise of 25% from the baseline value.^13^ Estimated glomerular filtration rate was calculated using the CKD epidemiology collaboration formula.^14^ Risk for CIN was calculated using the Mehran score.^15^ The study was approved by the institutional review board (approval number 2023‐421).

### 2.2 Statistical analysis

Categorical variables were reported as numbers and percentages, and continuous variables were reported as means and standard deviations or median and interquartile range. Continuous variables were tested for normal distribution using histograms and *Q*–*Q* plots.

## 3 RESULTS

The cohort consisted of eight inpatients with AKI who required coronary angiography. The mean age was 57 (±16) years and half were females. All patients had CKD with a mean baseline estimated glomerular filtration rate of 34 (±17) mL/min/1.73 m^2^. Additional baseline characteristics are presented in Table 1. Mean sCr at admission was 3.3 (±1) mg/dL, and before angiography, 3 (±1) mg/dL.

**Table 1:** Baseline characteristics.

	*n* = 8
Age, years (SD)	57 (16)
Female	50 (4)
Body mass index (SD)	31.9 (5.4)
Prior coronary artery disease	25 (2)
Prior coronary artery bypass graft	12.5 (1)
Prior cerebrovascular accident	25 (2)
Congestive heart failure	25 (2)
Left ventricular ejection fraction, % (SD)	50 (15)
Hyperlipidemia	37.5 (3)
Diabetes mellitus	37.5 (3)
Hypertension	87.5 (7)
Atrial flutter/fibrillation	25% (2)
Chronic obstructive pulmonary disease	12.5 (1)
Chronic kidney disease	100 (8)
Creatinine baseline, mg/dL (SD)	2.3 (0.8)
Estimated glomerular filtration rate baseline, mL/min/1.73 m^2^ (SD)	34 (17)
Hemoglobin, g/dL (SD)	11.6 (2.6)
Hematocrit, % (SD)	34 (6.8)
% (*n*) unless otherwise specified	

Abbreviation: SD, standard deviation.

Procedural characteristics are presented in Table 2. Indications for coronary angiography included work‐up for heart failure with reduced ejection fraction (*n *= 1), acute coronary syndrome (*n* = 3), pre‐transcatheter aortic valve replacement work‐up (*n* = 1), and pre‐renal transplant work‐up (*n* = 3). A clinical correlation was observed in those with heart failure, while in the reminder of patients AKI was found in routine up lab results. Mean contrast volume used was 14 (±4) mL with contrast volume to estimated glomerular filtration rate ratio of 0.7 (±0.4).

**Table 2:** Procedural characteristics.

Indication	
Pre‐renal transplant	37.5 (3)
Acute coronary syndrome	37.5 (3)
Pre‐transcatheter aortic valve replacement work‐up	12.5 (1)
Heart failure with reduced ejection fraction	12.5 (1)
Shock	0 (0)
Access	
Radial	37.5 (3)
Femoral	62.5 (5)
Pre‐hydration, median mL (IQR)	0 (0–137)
Post‐hydration, median mL (IQR)	125 (0–475)
Creatinine on admission, mg/dL (SD)	3.3 (1)
Creatinine before angiography, mg/dL (SD)	3 (1)
Contrast, mL (SD)	14 (4)
Contrast volume/estimated glomerular filtration rate	0.7 (0.4)
Contrast‐induced nephropathy risk score, (SD)	10.6 (4.1)
Contrast‐induced nephropathy risk, % (SD)	24 (15)
Instantaneous wave‐free ratio	12.5 (1)
Intravascular ultrasound	25 (2)
Further acute kidney injury during admission	0 (0)
Any increase in creatinine during admission	12.5 (1)
% (*n*) unless otherwise specified	

Abbreviations: IQR, interquartile range; SD, standard deviation.

**Figure 1:**
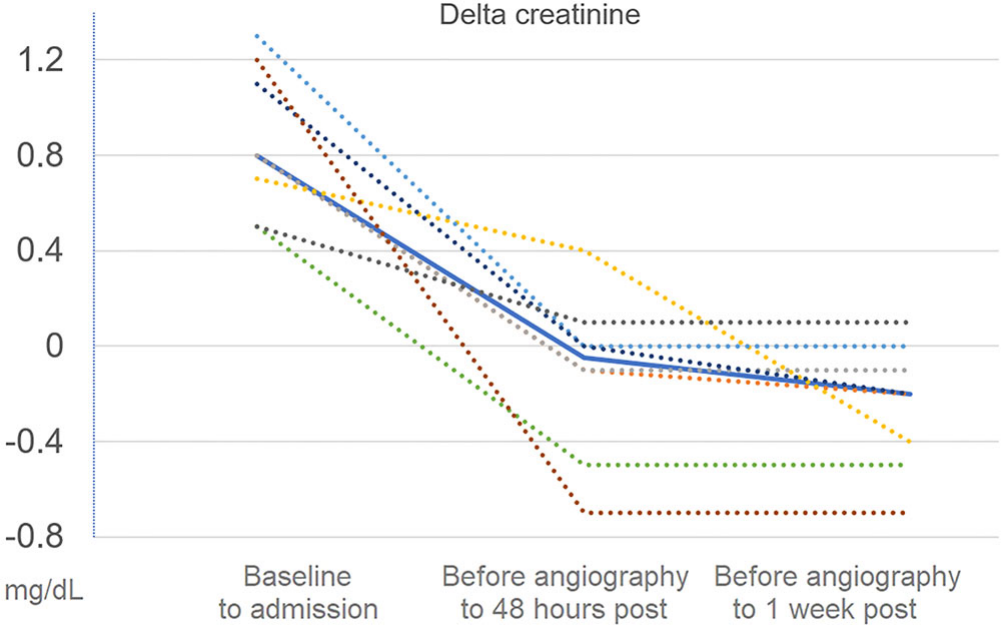
Change in creatinine before angiography to 48 h and 1 week after the procedure. Dotted lines depict each patient; The solid line depicts the mean.

One patient had a 0.1 mg/dL increase in creatinine during admission, and no other patient had worsening AKI. Figure 1 shows the mean change in creatinine before angiography to 48 h and 1 week after the procedure. Of note, among the 24 patients with AKI who did not receive ultra‐low contrast coronary angiography during the study period there was 16.7% further AKI, and 25% had further increase in creatinine during the admission.

## 4 DISCUSSION

The current case series is the first study to evaluate the safety of ultra‐low contrast coronary angiography in patients with pre‐existing AKI and a stabilized serum creatinine level. None of the patients developed CIN. Moreover, there was only a minimal rise in sCR in one patient.

Developing CIN is uncommon among all contrast agent exposed patients, but can surpass 30% in high risk populations.^1^ The term CIN, however, is misleading because there are causes for AKI after coronary angiography other than contrast agent exposure. Hypotension due to cardiac shock or other types of shock can lead to renal hypoperfusion; Diuretic or other nephrotoxic medication exposure with or without overdiuresis is common in cardiac patients. Cardiorenal synrdome is also a cause for AKI.^12^ Finally, additional coincidental etiologies of AKI that occur concomitentenly to coronary angiography could be the cause of peri‐procdural AKI, and still fall under the definition of CIN. A relatively small amount of contrast agent exposure alone is much less likely to inflict AKI. Renal blood flow accounts for approximately 20% of the cardiac output. During coronary angiography, contrast material is diluted by the time it reaches the kidneys. In a scenario when contrast is administered in small amounts such as 10 mL, only 1 mL would reach each kidney per minute. Even if the contrast material is not filtered out and 5 mL remains in each kidney, it is unlikely to impact the global renal function, as reflected by serum creatinine. Accordingly, none of the patients in the current study showed worsening renal function. Moreover, even low‐contrast percutaneous coronary intervention was previously shown to be feasible and safe.^11^, ^16^

Previous studies showed the feasibility of coronary angiography in patients with CKD^10^, ^11^, ^17^ and mean eGFR as low as 16 mL/min/1.73 m^2^.^10^, ^11^, ^17^ Median contrast administered was 8.8–13.5 mL^10^, ^11^ even among patients with prior coronary artery bypass grafts.^17^ Nonetheless, in these studies, patients had reduced but stable renal function. Within the limitation of the cohort's sample size, the current data suggest that even urgent, non‐emergent indications for coronary angiography can be safely performed on patients with AKI and a stabilized serum creatinine level.

Notably, although hospital stay is influenced mostly by the main problem since there was no further AKI in any of the patients, prevention of further deterioration of renal function by using ultra‐low contrast techniques potentially prevented delay in discharge. Nevertheless, ultra‐low contrast coronary angiography techniques are practical in a relatively stable population since they may be more time‐consuming and, therefore, may not apply to those who require urgent procedures.

There are limitations to the study that must be regarded. This is a retrospective study that could be impacted by selection bias. Additionally, the cohort was of small size. Conclusions relevant for a widespread population cannot be safely drawn and a larger prospective study is required to confirm the current results.

To conclude, the current data suggest that coronary angiography can be safely performed on patients with pre‐existing AKI and a stabilized serum creatinine level. The study should be viewed as hypothesis‐generating. Due to the cohort's small size and retrospective nature further studies are required to validate the results.

## CONFLICT OF INTEREST STATEMENT

The authors declare no conflict of interest.

## Data Availability

The data that support the findings of this study are available on request from the corresponding author pending approval of the institutional ethics committee. The data are not publicly available due to privacy or ethical restrictions.
